# Unveiling the Chemical Composition and Biofunctionality of *Hericium* spp. Fungi: A Comprehensive Overview †

**DOI:** 10.3390/ijms25115949

**Published:** 2024-05-29

**Authors:** Elizabeth Kostanda, Sanaa Musa, Idan Pereman

**Affiliations:** 1 Molecular Biology and Analytics of Medicinal Mushrooms Laboratory, Migal-Galilee Research Institute, Kiryat Shmona 11016, Israel; 2 Department of Biotechnology, Tel-Hai Academic College, Kiryat Shmona 11060, Israel; sanaa@migal.org.il; 3 Natural Compounds and Organic Synthesis Laboratory, Migal-Galilee Research Institute, Kiryat Shmona 11016, Israel

**Keywords:** *Hericium*, secondary metabolites, fruiting bodies, mycelia, polysaccharides, bioactive compounds, neurotrophins

## Abstract

In recent years, research on mushrooms belonging to the *Hericium* genus has attracted considerable attention due to their unique appearance and well-known medicinal properties. These mushrooms are abundant in bioactive chemicals like polysaccharides, hericenones, erinacines, hericerins, resorcinols, steroids, mono- and diterpenes, and corallocins, alongside essential nutrients. These compounds demonstrate beneficial bioactivities which are related to various physiological systems of the body, including the digestive, immune, and nervous systems. Extensive research has been conducted on the isolation and identification of numerous bioactive chemicals, and both in vitro and in vivo studies have confirmed their antimicrobial, antioxidant, immunomodulatory, antidiabetic, anticholesterolemic, anticancer, and neuroprotective properties. Therefore, this review aims to provide a comprehensive summary of the latest scientific literature on the chemical composition and secondary metabolites profile of *Hericium* spp. through an introduction to their chemical characteristics, speculated biosynthesis pathways for key chemical families, potential toxicological aspects, and a detailed description of the recent updates regarding the bioactivity of these metabolites.

## 1. Introduction

**Figure 1:**
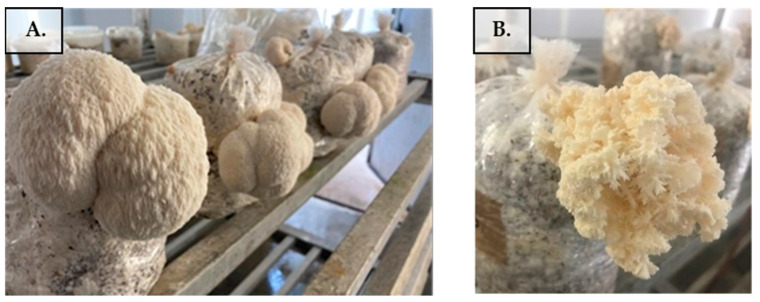
Controlled cultivation of *Hericium erinaceus* (**A**) and *Hericium coralloides* (**B**) at Pereman’s laboratory facilities—Migal.

The genus *Hericium* belongs to the Hericiaceae family, order Russulales, and class Agricomycetes. It includes about 15 species, which are distinguished macroscopically by the presence of branched vs. unbranched hymenophore structures supporting spines of various lengths, occurrence in single vs. multiple clumps, and microscopically by the presence of amyloid ornamented basidiospores [1]. The species *Hericium erinaceus* (Figure 1A), also known as Lion’s mane, has gained the most attention since its early documentation in traditional oriental medicine hundreds of years ago in the “Compendium of Materia Medica” written during the Ming dynasty in the 16th century. While this UNESCO’s heritage text mostly emphasizes the mushroom’s benefits relating to the digestive system, a research study in the early 1990s exhibited its neurotrophic functionality [2]. This pioneering study has stimulated consecutive studies which further demonstrated *H. erinaceus’s* neuroprotective and neurotrophic activities [1,3]. In addition, as part of the rising worldwide interest in medicinal mushrooms and their plausible health-promoting traits, the cognitive benefits of *H. erinaceus* were tested in clinical trials, further demonstrating a general trend of improvement in mental states [4,5]. The initial metabolic profiling of *H. erinaceus* has focused on erinacines and hericenones which are limited mainly to the mycelium and the sporocarp (fruiting body), respectively. Though these metabolites belong to terpenoid distinctive chemical groups (cyathane-type diterpenes and benzyl alcohol derivatives, respectively), both display biological functionalities with common characteristics. The cyathane diterpene chemical family found in the *Hericium* genus [2] was also found independently in mushrooms belonging to other genera like *Sarcodon*, *Cyathus*, and *Phellodon* [6,7,8]. Moreover, it was shown that most of the erinacine metabolites are synthesized by enzymes whose correlative genes are located adjacently in a conserved cluster. Interestingly, this conserved homology, which points towards a biological functionality, has motivated the reconstruction of the cyathane diterpene pathway in yeast and resulted in the synthesis of “non-natural” erinacine analogs some of which exhibit biofunctionality [9].

While *H. erinaceus* has been the main target for studies performed within the *Hericium* genus, additional species have also been analyzed for their distinctive metabolic profile and possible health-promoting functionalities [10]. These include *H. coralloides* known as the Coral tooth fungus [11,12] (Figure 1B); *H. americanum*, known as the Bear’s head tooth fungus and located in North America (initially identified as *H. coralloides* and later defined as a separate species) [13]; *H. flagellum* which is associated with conifers and has been preliminarily analyzed only in recent years [14]; and *H. Rajendrae* which is native to the Himalayas [2].

The ongoing discovery of new species and the analysis of their metabolomes results in accumulating valuable data of phytochemicals belonging to different chemical families (for additional information, recent reviews thoroughly outline the cumulative knowledge regarding some of the main chemical groups in *Hericium* spp. and their biological functionalities) [10,15,16,17]. In turn, this data inflation concerning potentially bioactive compounds in *Hericium* spp. specifically and medicinal mushrooms in general sets a great challenge in understanding the underlying molecular mechanisms and the overall interplay between these pathways leading to a specific physiological phenotype. One of the developing research approaches which is designated to resolve this complexity is the use of Quantitative Structure–Activity Relationship (QSAR) models. This computational strategy can screen big-data phytochemical libraries and their interactions with specific targets, while incorporating machine learning which uses known drugs as a reference guideline. For example, a recent study employing QSAR has indicated a possible acetylcholinesterase inhibition which is mediated by erinacerin A and hericenone B [18].

In the following review, we aim to summarize the most updated literature regarding the *Hericium* genus. We have addressed the unique metabolic profile characterizing *Hericium* species while referring to the chemical composition of polysaccharides and key secondary metabolite groups, including corallocins, erinacerins, hericioic acids, hericenes, hericenones, hericerins, erinacines, and other specialized metabolites. We have further explored the speculated biosynthesis pathways for key chemical families. Finally, we have addressed the correlation between the different chemical groups and their corresponding biofunctionality while referring to some of their documented toxicological aspects.

## 2. Toxicology Studies

While numerous studies have confirmed the remarkable therapeutic properties of *Hericium* spp. mushrooms, it is crucial to comprehensively evaluate their safety profile and potential risks associated with their use. Experimental analyses focusing on the action of *H. erinaceus* mycelium components suggest their safety and lack of side effects [19,20]. For instance, Chen et al. conducted sub-chronic toxicity and genotoxicity studies of an *H. erinaceus* β-glucan extract preparation in Sprague Dawley rats. The results observed no treatment-related significant toxicological effects in various parameters, including clinical observations, ophthalmic examinations, body weight gains, feed consumption, organ weights, hematology, serum chemistry, urinalysis, and terminal necropsy findings. Based on the sub-chronic study, the No Observed Adverse Effect Level (NOAEL) for *H. erinaceus* β-glucan extract preparation was determined as 2000 mg/kg body weight per day, which was the highest dose tested [21]. Similarly, Li et al. demonstrated that the consumption of standardized erinacine A-enriched *H. erinaceus* mycelium poses no genotoxic hazards to individuals, as evidenced by in vitro mutagenicity tests and in vivo assays (mouse erythrocyte micronucleus assay) [22]. Moreover, Lew and co-workers explored the neuroprotective effects of an *H. erinaceus* standardized aqueous extract (HESAE) against high-dose corticosterone-induced oxidative stress in rat pheochromocytoma (PC12) cells, a cellular model mimicking depression. Their findings revealed that HESAE demonstrated a neuroprotective effect via the upregulation of antioxidant enzyme activities in an in vitro model of depression [23]. In another study published by Lakshmanan et al., the potential toxic effects of the aqueous extract from the fruiting bodies of *H. erinaceus* were evaluated in rats through a sub-chronic oral toxicity study. The researchers observed no mortality or morbidity in any treated or control rats. The results indicated that the daily oral administration of HEAE at three different doses for 90 days did not elicit adverse effects on general behavior, body weight, hematological parameters, clinical biochemistry, or relative organ weights. Histopathological examination at the end of the study revealed normal tissue architecture, with only a few non-treatment-related histopathological changes observed in the liver, heart, and spleen [24].

A study conducted by Li et al. investigated the efficacy and safety of three *H. erinaceus* mycelia (EAHE) capsules, which contained erinacine A as the active ingredient, in treating patients with mild AD. Their findings revealed that after 49 weeks of treatment, patients receiving EAHE exhibited improvements in cognitive abilities by the higher screening instrument, mini-mental state examination, and instrumental activities of daily living scores compared to those in the placebo group. Importantly, the study concluded that is safe, well-tolerated, and may be important in achieving neurocognitive benefits [25].

## 3. Identification of Novel Bioactive Compounds in *Hericium* spp. and Their Biological Functions

*Hericium* species exhibit an exceptional abundance of structurally diverse bioactive components found within both their fruiting bodies and mycelia [26]. It is worth noting that the chemical composition can vary depending on cultivation methods and natural habitats. For instance, studies by Guan et al. revealed that artificially cultivated *H. coralloides* fruiting bodies include approximately 33% polysaccharides, 16% crude protein, 12% total ash, 8% reducing sugars, 6% crude fat, 5% crude fiber, 3% total triterpenoids, 1% total saponins, 0.88% total flavonoids, and 0.5% total alkaloids. The total sterol and phenol contents were also measured at 0.43% and 0.18%, respectively [11,27]. In contrast, Cohen et al. previously demonstrated that the fruiting bodies of *H. erinaceus* contain approximately 61.1% total carbohydrate and 20.8% protein, while the mycelia biomass was found to have about 43.9% total carbohydrate and 42.5% protein [28]. Yang et al. identified about 102 compounds from the fruiting bodies of *H. erinaceus*, including organic acids, nucleotides and analogues, amino acids, carbohydrates and derivatives, flavonoids, unsaturated fatty acids, terpenoids, phenolic acids, phenylpropanoid, and steroids [29]. The authors further suggested that flavonoids which were qualitatively detected in the analysis (luteolin, apigenin, diosmetin, hispidulin, and acacetin) could function as active components in *H. erinaceus* based on previous studies which demonstrated flavonoids’ pharmacological functions which include neuroprotection, improved learning and memory, anti-inflammatory, and more [30,31,32]. Of note, in a study by He et al., two isoflavones were identified and isolated in *H. erinaceus* mycelium: genistein and daidzein, comprising 15% and 12% of the ethanolic extract, respectively [33]. Moreover, the *Hericium* genus is abundant in bioactive chemicals like polysaccharides, alkaloids, hericenones, erinacines, hericerins, resorcinols, steroids, mono- and diterpenes, and corallocins [16,17,20,26,29,34,35,36]. Extensive research underscores the therapeutic potential of *Hericium* fungi, demonstrating notable antioxidative, anti-inflammatory, antimicrobial, antidiabetic, and anticancer properties. Additionally, these fungi exhibit promising effects in treating cognitive disorders, Alzheimer’s disease, ischemic strokes, Parkinson’s disease (PD), depressive disorders, and age-related impairment [10,37,38,39,40]. In the current manuscript, we will provide an overview of the chemical nature and identification of both known and novel compounds that could potentially be responsible for the observed health-promoting properties. Furthermore, the measured percentage concentration of key metabolites will be indicated when available.

The composition of secondary metabolites can be significantly affected by external environmental variations and growth conditions [41,42]. Numerous recent studies have focused on examining the correlation between mushroom cultivation parameters, mainly substrate composition, and the consequential modification of the metabolomic profile. In these studies, different ratios of the base substrate (such as hard-wood sawdust) vs. various supplements (such as cottonseed hulls, olive press cake, and rice burn) were tested. In these analyses, the synthesis of target beneficial bioactive compounds was measured following cultivation in the different substrate compositions with the goal of enhancing the compound’s concentration. For example, Turk et al. demonstrated that hericene A significantly increases from 1.93 to 3.46 mg/g dry *H. erinaceus* when specific agricultural by-products are included in the substrate. Additionally, they found that the incubation days for fruiting bodies affected the hericene A content, whereas there was little effect on mycelia growth periods [43]. Similarly, Atila et al. have tested the effect that various agricultural wastes in the substrate have on the mushroom’s fruitbody antioxidant activity and phenolic composition [44]. A second approach involves an undirected metabolome analysis under different cultivation regimes. For example, in a collaborative study, questioning the correlation between cultivation characteristics and resulting mushroom metabolome, we observed that changes in the concentrations of olive mill solid waste (OMSW) in the substrate dramatically affected the composition of mushroom-specialized metabolites. Interestingly, OMSW led to an increase in the beneficial hericenone family in *H. erinaceus* fruiting bodies, as well as several erinacerins in the mycelium, alongside a reduction in the abundance of toxic enniatin metabolites [45].

### 3.1. Polysaccharides

Polysaccharides derived from the *Hericium* spp. have been of particular interest due to their attributed bioactivity [46]. These polysaccharides are primarily located in the cell wall and constitute a significant portion of both the fruiting bodies and mycelium biomass [20]. Yan et al. demonstrated that extracting water-soluble polysaccharides from *H. erinaceus* fruiting bodies using various solvents significantly affected the extraction yields, molecular weight, monosaccharides compositions, preliminary structural characteristics, and microstructures of the polysaccharides. Their study also revealed that citric acid solution extraction exhibited superior antioxidant properties compared to hot water and 0.9% NaCl extracts in in vitro assays. Furthermore, they observed that the citric acid extracts exhibited the strongest inhibitory effects on the α-glycosidase and α-amylase activities [47]. Hu et al. purified and analyzed a polysaccharide fraction from the fermented mycelium water extract of *H. erinaceus*, which they named PHEB. Their analysis revealed that PHEB exhibits a uniform single peak, indicating its high degree of homogeneity. The mass-average molecular mass of PHEB is measured at 36.1 kDa. They also identified four main monosaccharide components: D-galactose (Gal), D-glucose (Glu), D-mannose (Man), and D-glucuronic acid (GlcUA). Their study revealed that a six-week PHEB administration significantly improved the cognitive behavior of mice. Brain injury, amyloid-beta deposition, and tau hyperphosphorylation were alleviated in PHEB-treated AD mice without changes in other tissues. PHEB alleviated the oxidative stress in the brains of AD mice via regulation of Nrf2 and its downstream kinase, which further improved the cholinergic system function. Proteomics and bioinformatics analysis showed that the therapeutic effect of PHEB is achieved by regulating calcium homeostasis mediated by oxidative stress. Furthermore, PHEB regulated the CaMK II/IV to achieve calcium homeostasis in the brain and ultimately show anti-AD properties [48].

Wu et al. described a novel heteropolysaccharide fraction (HEP-W) with high immunomodulatory activity that was isolated from the fruiting bodies of *H. erinaceus* using hot water. Structural characterization revealed that HEP-W had an average molecular weight of 1.59 KDa and was composed of rhamnose (Rha), fucose (Fuc), mannose, glucose, and galactose at a molar ratio of 0.98: 1.59: 0.89: 5.60: 7.06. The main glycosidic linkage types of HEP-W consisted of (1→)-α-D-Glc, (1→3,6)-α-D-Glc, (1→2,6)-α-D-Gal, T-β-Gal, (1→3,4)-β-D-Man, (1→3)-α-Rha, and (1→2)-β-L-Fuc by periodate oxidation–Smith degradation and NMR analysis. The bioactivity tests showed that HEP-W could significantly promote the pinocytic and phagocytic capacity and increase the nitric oxide (NO), interleukin-6 (IL-6), and tumor necrosis factor-alpha (TNF-α) secretion by activating corresponding mRNA expression in macrophages (RAW 264.7 cells) through MyD88/IR AK-1/TRAF-6/PI3K/AKT/MAPKs signaling pathways [49].

Recently, Li and co-workers isolated a purified fraction of *H. erinaceus* polysaccharides (HEP) from the fruiting bodies. Initially, they utilized hot ethanol to eliminate monosaccharides and colored substances, followed by hot deionized water and chromatographic purification. They showed that the monosaccharide composition of this fraction consists of five kinds of monosaccharides, including mannose, rhamnose, glucose, galactose, and L-fructose, with the proportion of (0.8):(0.1):(9.7):(10):(1.6). Glucose content was the highest, while the rhamnose content was the lowest. They showed that the pretreatment of IPEC-J2 cells with HEP had a protective effect against oxidative stress (mediated by H_2_O_2_) by reducing apoptosis via mitochondrial and death receptor pathways [50].

Zhang et al. extracted polysaccharides from *H. coralloides* by different methods, including heat reflux (HRE-P), acid-assisted (ACE-P), alkali-assisted (AAE-P), enzyme-assisted (EAE-P), ultrasonic-assisted (UAE-P), cold water (CWE-P), pressurized hot water (PHE-P), hydrogen peroxide/ascorbic acid (HAE-P) system, and acid-chlorite delignification (ACD-P) methods. The experimental outcomes indicated notable variations in the extraction yields, chemical compositions, monosaccharide constituents, and molecular weights of the obtained nine polysaccharides but similar Fourier-transform infrared (FT-IR) and nuclear magnetic resonance (NMR) spectra characteristics. HRE-P had the second highest yield, the highest polyphenol content, and the biggest Mw compared to the other polysaccharides and demonstrated the highest activity against 2,2′-Azino-bis(3-ethylbenzothiazoline-6-sulfonic acid) (ABTS) and OH radicals. In comparison, CWE-P demonstrated the highest activity against ABTS, 2,2-Diphenyl-1-picrylhydrazyl (DPPH), and superoxide radicals, and UAE-P against DPPH radicals. In addition, UAE-P, CWE-P, and HAE-P exhibited better protective effects on L929 cells when compared to the other obtained polysaccharides. Additionally, correlation analysis indicated that the monosaccharide composition and total polyphenol content were two prominent variables influencing the bioactivity of *H. coralloides* polysaccharides [51].

Another study conducted by Li et al. aimed to evaluate the effects of polysaccharides extracted from *H. erinaceus* fruiting bodies (HEFPs) on the inflammatory response to oxidative stress in a mouse model of ulcerative colitis (UC) induced by dextran sodium sulfate ingestion. Their analysis revealed that the polysaccharides fraction had a peak molecular weight, average molecular weight, and number average molecular weight of 17.2 kDa, 16.3 kDa, and 15.9 kDa, respectively. HEFPs were found to consist of six monosaccharides, namely fucose, glucosamine, galactose, glucose, xylose, and mannose, with a molar ratio of (0.98):(0.009):(0.0476):(0.369):(0.004):(0.043). The study demonstrated that HEFPs effectively alleviated UC-related symptoms, reduced associated histopathological damage, upregulated antioxidant enzymes, and mitigated inflammation by targeting the NLRP3 inflammasome/Caspase-1 pathway. Additionally, HEFPs were shown to restore the perturbed gut microbiota associated with UC [52].

### 3.2. Corallocins 

**Figure 2:**
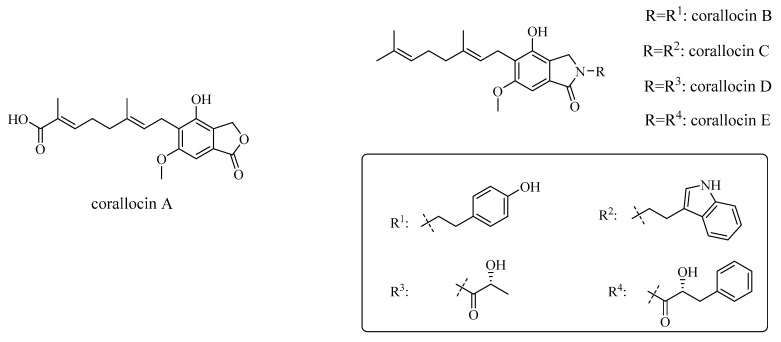
Chemical structures of corallocins A–E.

The corallocins represent a group of benzofuranone- and isoindolinone-type meroterpenoids. Wittstein et al. isolated three corallocins, namely A (2.8 mg), B (29.4 mg), and C (3.4 mg), from 7.88 g ethyl acetate crude extract of fruiting bodies of *H. coralloides* [53], while Ryu et al. isolated corallocins from the fruiting bodies of *H. erinaceus* [54]. The chemical structures of these compounds were elucidated through various spectral methods, including the interpretation of 1D- and 2D-NMR, HR-ESIMS and FTIR analyses [53]. Subsequently, the same research group discovered two additional unprecedented isoindolinone derivatives, named corallocins D and E. The purification of total extract was achieved using a preparative reverse-phase liquid chromatography, yielding 1 mg and 0.7 mg of corallocin C and D, respectively, from 272 mg of ethyl acetate crude extract. The structures of these compounds were elucidated using HR-ESIMS and NMR analyses. Through electronic circular dichroism (ECD) experiments, the absolute configuration of both compounds was determined to be the R configuration, as they share a similar sole chiral center and exhibit close positive ${\left[\alpha \right]}_{D}^{20}$ values (+24 and +26, respectively) [55]. Corallocins B–E share the same isoindolinone structure with a different substitution at N-2, while corallocin C is distinguished as a rare indole isoindolinone derivative. Conversely, corallocin A is characterized by a benzofuranone-type meroterpenoid structure, featuring a carboxylic acid group located on other side of a geranyl moiety (Figure 2).

The neurotrophic potential of three isolated compounds from *H. coralloides*, corallocins A, B, and C, to induce rat pheochromocytoma cell line (PC12) differentiation was analyzed via stimulation of the 1321N1 astrocytoma cell line [53]. Corallocin A and C were able to upregulate nerve growth factor (NGF) expression in astrocytoma cells and neurite outgrowth in PC12 cells. Corallocin B and C upregulated brain-derived neurotrophic factor (BDNF) expression as well. Interestingly, corallocin A, isolated from *H. erinaceus*, exhibited similar activity in another study, inducing NGF protein expression in C6 glioma cells [54]. In another study, corallocin A was isolated from *H. erinaceus* and exhibited the ability to elicit strong survival and neurotrophic responses on hippocampal neurons’ (DIV3) culture in the absence of serum supplementation [56]. Additionally, corallocins D and E were assessed for their cytotoxic and antimicrobial effects in vitro, showing weak to moderate cytotoxicity against HeLa cells (KB 3.1), *Mucor hiemalis,* and *Bacillus subtilis* [55].

### 3.3. Erinacerins

**Figure 3:**
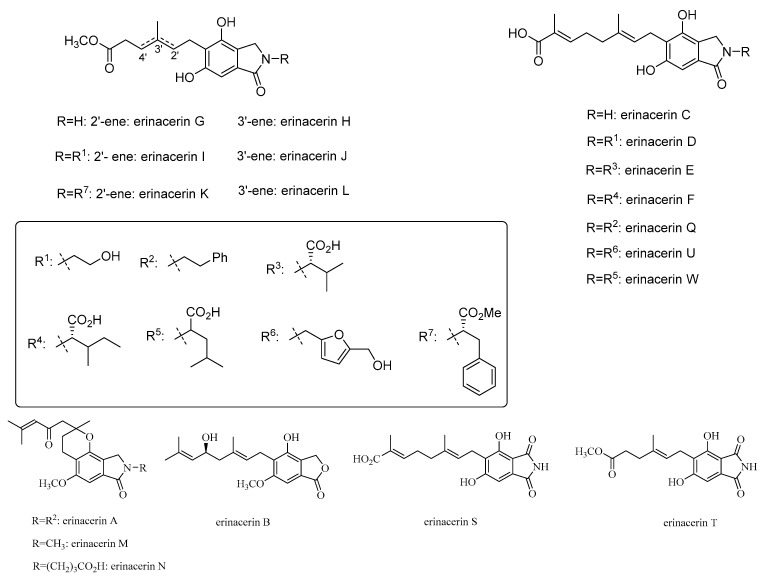
Chemical structures of selected erinacerins.

The isoindoline-1-ones represent a major group of secondary metabolites found within the *Hericium* genus. Figure 3 illustrates the chemical structures of selected erinacerins. Wang et al. isolated and characterized ten isoindolin-1-ones, named erinacerins C–L, from the solid culture of *H. erinaceus* [57]. These compounds’ structures were determined using spectroscopic methods, with the absolute configurations of some assigned by comparing their specific rotations with those of related phthalimidines. In their study, the absolute configurations of erinacerins E, F, K, and L were determined through the synthesis of phthalimidines with L- and D-amino acids, which were deduced to have negative specific optical rotations, indicating the S-configuration.

Lin et al. isolated a new compound with an isoindoline-1-one skeleton from the mycelia of *H. erinaceus*, named erinacerin W [58]. The purification of this compound was accomplished using silica gel chromatography followed by reversed-phase chromatography, yielding about 13 mg from 250 g of freeze-dried mycelia of *H. erinaceus*. The structure elucidation was conducted based on an analysis of the MS and NMR spectral data. However, in their study, they did not determine the absolute configuration or specific rotation. Based on previous data regarding erinacerin F, which feature an isoleucine in the side chain, and considering that erinacerin W contains leucine at N-2, it is reasonable to assume that it shares a similar absolute S configuration.

Erinacerins A, M, and N were isolated from the fruiting bodies of *H. erinaceus* by Ashour et al., and their structural elucidation was confirmed based on spectroscopic studies. These compounds contain an isoindoline-1-one moiety along with a cyclic ether. They differ in their substitution at N-2, and they are present as a racemic mixture [59]. Previously, Wang et al. isolated erinacerins Q, S, and T from a solid culture of *H. erinaceus* and identified their chemical structure using spectroscopic methods. The latter two compounds are suggested to be based on a phthalimide skeleton [60]. In the same study, two additional compounds classified as alkaloids were isolated, termed erinacerin N and M. This nomenclature contrasts with the study by Ashour et al., where compounds bearing the same names exhibit entirely distinct structures [59,60].

Several erinacerins have been found to possess inhibitory effects on α-glucosidases, suggesting their potential as antidiabetic agents [57,60,61]. Wang et al. demonstrated that erinacerins Q, S, and T exhibit inhibitory activities against both protein tyrosine phosphatase-1B (PTP1B) and α-glucosidase [60]. Erinacerins M and N were evaluated for their cytotoxic effect against six human cancer cell lines (SH-SY5Y, 1321N1, HCT-116, Caco-2, OVK18, and HeLa), showing moderate activities across different cell lines [59]. The newly discovered erinacerin W showed protective potential against inflammation-associated neurotoxicity. At a concentration of 20 μg/mL, erinacerin W exhibited protection against the excessive expression of the pro-inflammatory cytokines IL-6, IL-1β, and TNF-α in SH-SY5Y cells, induced by lipopolysaccharide-activated BV-2 microglia cells, thus indicating its potential in attenuating the progression of neurodegenerative diseases [58].

### 3.4. Hericioic Acids

**Figure 4:**
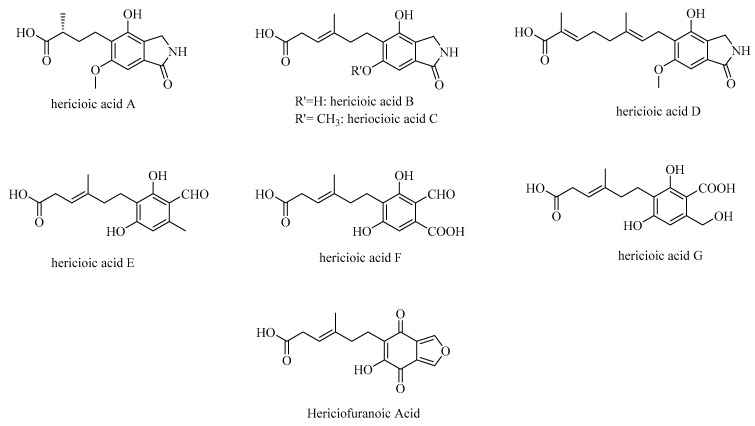
Chemical structures of hericioic acids A–G and hericiofuranoic acid.

Recently, Sum and co-workers isolated eight new isoindolinone and benzofuranone-type derivatives from the rare European edible mushroom *H. flagellum*. The ethyl acetate extract was purified using a preparative HPLC system. The authors named the novel compounds hericioic acids A–G and hericiolfuranoic acid [62]. They succeeded in isolating 6.9 mg, 6.8 mg, 11.6 mg, 23.3 mg, 1.4 mg, 8.5 mg, 1.7 mg, and 2.9 mg of hericioic acids A–G and hericiolfuranoic acid, respectively, from 0.6 g of mycelial extracts and 6.4 g solid-state fermentation extracts. The chemical structures were successfully elucidated based on the interpretation of extensive 1D- and 2D-NMR measurements coupled with HR-ESIMS analyses (Figure 4). To elucidate the absolute configuration of hericioc acid A, its ECD spectrum was measured and compared to the calculated ECD spectra of both R and S isomers. The measured and calculated ECD spectra of the R-isomer revealed a close accordance over the whole range that unambiguously assigns the stereogenic center to be in R configuration.

*H. flagellum*-isolated compounds were tested in PC12 cells for their neurotrophic activity (alongside the addition of 5 ng NGF). All compounds were shown to promote neurite outgrowths, with hericioic acid C, D, and hericiofuranoic acid exhibiting the most potent effects. Their cytotoxic effect was tested as well; none of the compounds exhibited significant growth inhibition against sensitive mouse fibroblast cells (L929) or human endocervical adenocarcinoma (KB 3.1) [62].

### 3.5. Hericenes, Hericenones and Hericerins

Hericenes are classified as geranyl-resorcinols, containing a resorcinol and geranyl unit. To date, over seventy geranyl-resorcinols have been isolated and identified from the fruiting bodies of the *Hericium* genus [63]. Arnone and co-workers were the first to isolate and elucidate hericenes A–C, characterized by the presence of three different fatty acids [64]. Later, Ma et al. isolated hericene D, which contains linoleic acid as one of the fatty acids. The authors conducted methanolysis of hericene D and confirmed the production of methyl ester of linoleic acid, identified by GC-MS [65].

Hericenones, on the other hand, are oxidation products on the geranyl side chain of hericenes, likely produced from oxidative metabolism processes. Their content has been reported to be around 2.36 mg/g dry weight of *H. erinaceus* fruiting bodies. Historically, the first isolation report on hericenones A and B was conducted by Kawagishi et al. in 1990, followed by the isolation and identification of hericenones C-H [2,66,67]. The authors described the structures of hericenones A and B containing a phthalide nucleus with a carbonyl group at the benzylic position located ortho to the phenolic hydroxyl group [68]. However, two years later, Rama Rao et al. reported the first total synthesis and revised structures of hericenone A and B. Through extensive NMR and IR analyses, they demonstrated that the carbonyl group at the benzylic position is located meta to the phenolic hydroxyl group, which fully matches those of the natural product [69]. Hericenones F–H, isolated from the fruiting bodies of *H. erinaceus*, have a chromene framework with long-chain fatty acids [67]. Additionally, hericenone L, an oxidation product of hericenone C at the aldehyde moiety, was isolated and identified by Ma et al. using spectral data. The fatty acid was confirmed to be palmitoyl through the methanolysis of hericenone L, as identified by GC-MS analysis [70].

**Figure 5:**
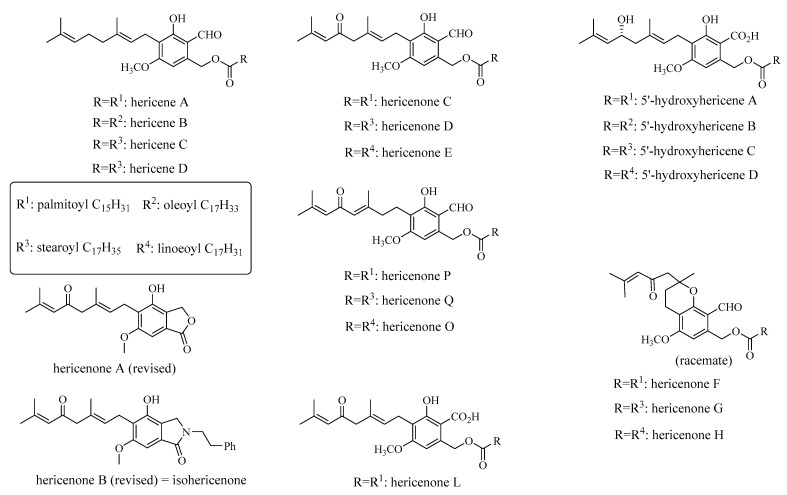
Chemical structures of selected hericene and hericenone derivatives.

Recently, Thongkongkaew and co-workers reported the isolation and structure elucidation of new compounds from the fruiting bodies of *H. erinaceus*, identified as 5′-hydroxyhericenes A–D existing as an inseparable mixture (4.7 mg from 5.9 g of hexanoic crude extract) [71]. Since attempts to separate these compounds by normal phase HPLC were unsuccessful, the structure of geranyl-resorcinol parts was elucidated by NMR and HRMS using the inseparable mixture. The fatty acids present in the compounds were identified as palmitic, stearic, oleic, and linoleic in a ratio of approximately 21:11:2:1 using GC-MS through a methanolysis approach. To determine the absolute configuration of the hydroxyl group, a modified Mosher’s method was employed. Accordingly, the absolute configuration was concluded to be R (Figure 5).

Hericerins are also among the members of the geranyl resorcylate family of natural products, isolated from *H. erinaceus*. Li et al. have isolated and identified a number of hericerin structures [63]. In 1991, Kimura and co-workers determined and published the chemical structure of hericerin [72]. However, subsequent research by Kobayashi et al. attempted to synthesize it, leading to a revision of the structure to be the carbonyl regioisomer [73].

**Figure 6:**
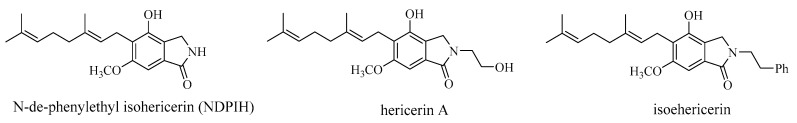
Chemical structures of selected hericerins.

Notably, the chemical structures of hericerins share the same chemical structure as corallocins B–E, with variations in substitution at N-2 (Figure 6).

Kawagishi and co-workers were the first to utilize the stimulation of NGF synthesis as a research method to assess the neurotrophic properties of hericenones C–E [2]. This seminal study stimulated the investigation of *Hericium* spp. metabolites as potential therapeutic compounds for the treatment of neurodegenerative diseases.

An *H. erinaceus*-isolated mixture of 5′-hydroxyhericenes A–D inhibited xanthine oxidase activity, while adenosine and hericenones C and D showed the scavenging potential of reactive oxygen species generated by xanthine oxidase. Hericerin, which was isolated as well, exhibited a strong growth inhibitory activity against T47D breast cancer cells and, to a lesser extent, against MDA-MB-231 breast cancer. Based on the apoptosis assay, it can be assumed that the growth inhibitory effect of hericerin may be mediated through nonapoptotic pathways [71]. In another analysis by Ruan et al., cytotoxic activity against cancer cells was tested using several benzaldehyde derivatives isolated from *H. erinaceus*. Hericenone Q showed moderate cytotoxic activity against a human colorectal carcinoma cell line (HCT-116) and a strong activity against a human liver cancer cell line (Hep-G2) [74]. A comparative investigation of the neuroprotective properties of *H. erinaceus*-isolated hericenone C and deacylhericenone (hericenone C treated with lipase), published by Shimizu, also demonstrated that the BDNF mRNA expression in human astrocytoma cells (1321N1) and the protection against H_2_O_2_-induced oxidative stress were considerably higher in the case of deacylhericenone [75]. Martínez-Mármol et al. published an elaborated work regarding the neurotrophic effects of *H. erinaceus*-isolated compounds and specifically hericerin derivatives in vitro and in vivo. N-dephenylethyl isohericerin (NDPIH), together with its hydrophobic derivative hericene A, were found to be highly potent in promoting extensive axon outgrowth and neurite branching in cultured hippocampal neurons even in the absence of serum, demonstrating potent neurotrophic activity. Mice fed with *H. erinaceus* crude extract and hericene A also exhibited increased neurotrophin expression and downstream signaling, resulting in significantly enhanced hippocampal memory [56].

### 3.6. Erinacines

**Figure 7:**
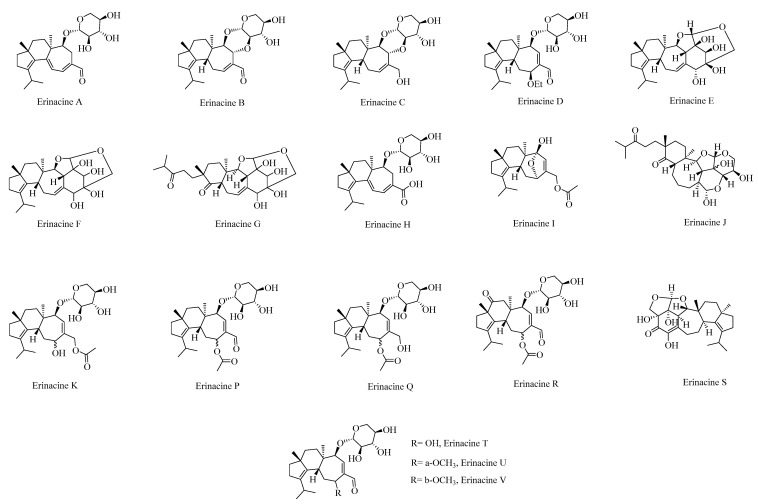
Chemical structures of erinacines A–K and P–V.

Erinacines represent a class of cyathane-type diterpenoids characterized by a five–six–seven tricyclic carbon scaffold, and they are considered major active agents isolated from the cultured mycelia of *Hericium* spp. [76]. To date, researchers have isolated and identified more than 30 erinacines; selected erinacines are illustrated in Figure 7 (erinacines A–K and P–V) [1,38,77,78,79]. These compounds are characterized by the presence of D-xylose in their scaffold, with the sugar determined to be in the β configuration. Chiu et al. quantified the contents of erinacine A, C, and Q in the mycelium of *H. erinaceus* to be 5 mg/g, 0.019 mg/g, and 0.094 mg/g dry weight, respectively. Chen et al. isolated erinacine S, a rare sesterterpene, from the mycelia of *H. erinaceus*. They purified and isolated it using Sephadex LH-20 and silica gel columns, obtaining erinacine S in 525 mg from 2 kg of the freeze-dried mycelia of *H. erinaceus*. The structure was elucidated by spectroscopic analysis. Furthermore, the exact structure was validated through X-ray analysis by acetylating it to yield diacetate-erinacine S, which was suitable for single-crystal X-ray analysis [80,81].

Zhang et al. extracted and isolated three new cyathane-diterpenes, erinacines T–V, from liquid cultures of *H. erinaceus*. The chemical structures were elucidated through comprehensive spectroscopic analysis, which included 2D-NMR and ESI-HRMS techniques. The distinction between erinacines U and V lies in the stereochemistry of the R-bond, and both compounds are confirmed to be the O-methylated cyathane diterpene derivatives of erinacine T [82]. Additionally, the latter is identified as the acylated derivative of erinacine P [83].

Erinacines are known to have the ability to affect neurons in various ways, which places them as novel agents in treating neurodegenerative disorders [84]. Many of their impacts are summarized in a recent review [85]. They have been identified as the primary stimulators of NGF biosynthesis in vitro in 1321N1 and PC12 cells. It has been demonstrated that erinacines A–H exhibit potent stimulating effects on NGF. Erinacine A, which has been extensively studied, has also been reported to demonstrate a strong enhancing effect on increasing the content of catecholamine and NGF in the central nervous system of rats [86,87]. Furthermore, a recent study found that an ethanolic extract enriched with erinacine A was able to repair scopolamine-induced AD patterns in damaged zebrafish brains. This repair was evidenced by improvements in memory evaluated through behavioral and biochemical tests on brain tissue [88].

In a new study, a new compound from the erinacine family, erinacine S, showed a promising effect on myelin repair and emotional abnormality reduction following demyelination in the corpus callosum of rats’ brain. Simultaneous administration of *H. erinaceus* mycelium extract and erinacine S, as well as delayed erinacine S treatment, preserved oligodendrocytes and myelin during chemical-induced acute demyelination by cuprizone. Interestingly, erinacine A was tested as well and did not display the same effect. Furthermore, post-administration of erinacine S in mouse models with acute or chronic cuprizone-induced demyelination not only inhibited demyelination and gliosis, but also alleviated anxiety and depression in both acute and chronic cuprizone-fed mice [81]. The role of erinacines in the amelioration of neurodegenerative disorders was analyzed in a Qi study based on a neurite outgrowth assay using a PC12 cell line and anti-inflammatory activities using a BV2 microglia cell line. Erinacines A, C, F, and L significantly promoted neurite outgrowth in PC12 cells upon the addition of NGF, in comparison to NGF alone. In addition, they significantly inhibited lipopolysaccharide (LPS)-induced NO production in BV2 microglia cell cultures with very low IC50 values. Among the four compounds, erinacine L showed the highest activity. In order to further investigate the molecular mechanisms mediating the observed NO inhibition, the authors implemented a computational approach simulating the docking of the active compounds with inducible NO (iNOS). In turn, this analysis supported erinacine L stronger inhibition activity due to its hemiacetal structure which mediates a lower docking energy value with iNOS [89].

Lee et al. showed that *H. erinaceous* mycelium and its ethanol extraction of erinacine A treatment exhibited dose-dependent preventive and therapeutic effects in restoring dopaminergic degeneration and alleviating motor dysfunctions. Their study revealed that post-treatment with erinacine A could reduce 1-methyl-4-phenylpyridinium (MPTP)-induced neurotoxicity via activation of the PAK1, AKT, LIMK2, MEK, and Cofilin survival pathways, as well as reductions in the IRE1α, TRAF2, ASK1, GADD45, and p21 cell death pathways in N2a neuron cells and in an MPTP model of PD. Thus, erinacine A could be used as a valuable neuroprotective and therapeutic agent, improving pathological conditions and behavioral deficits during PD treatment [40]. In another recent study, the antioxidative efficacy of micronized *H. erinaceus* (HEM), rich in erinacine A, against MPTP-induced damages in mice was evaluated. MPTP is a toxin used to induce PD in mice by generating oxidative stress. *H. erinaceus* micronized mycelium reversed the MPTP-induced dopamine reduction in the tested mice brain in a dose-dependent manner. Similarly, the effect of erinacine A-enriched HEM treatment was further tested on red blood cells’ oxidative stress enzyme biomarkers indicating that HEM administration could reverse the reduced enzyme activities in a dose-dependent manner. The same effect was seen analyzing protein carbonyl and malondialdehyde, oxidative stress biomarkers, in mice brain and liver, which displayed reduced levels of the two in the MPTP+HEM groups, compared to MPTP groups [90]. Wachiryah Thong-asa recently published a similar study analyzing mice with trimethyltin-induced neurodegeneration. *H. erinaceus* mycelia extracts, from different cultivating formulas, and thus with diverse erinacines composition, were given orally to mice subsequent to the toxin intake. The erinacine composition from EME/R (combination of eucalyptus and rubber wood in the substrate), containing erinacines A, C, D, G, H, I, K, and R, exhibited significant positive effects on spatial learning, memory, flexibility, and anxiety. These findings emerged concurrently with the significant mitigation of hippocampal lipid peroxidation, CA1 hippocampal, cortical neuron, and corpus callosum white matter degeneration [91].

### 3.7. Other Specialized Metabolites

#### 3.7.1. Lovastatin and Ergothioneine

**Figure 8:**
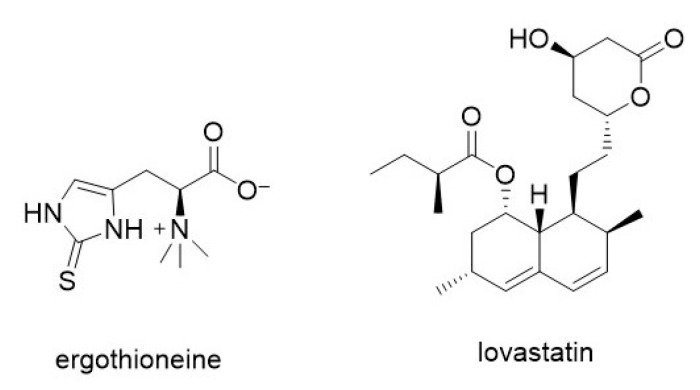
Chemical structures of ergothioneine and lovastatin.

Lovastatin and ergothioneine (also referred to as the “longevity vitamin”) have both been recognized as beneficial metabolites which can be found either in the mycelium or the fruiting body of mushrooms. In a study comparing biologically active substances, bioelements, and glucan content between the fruiting bodies and mycelia of *H. erinaceus*, *H. coralloides*, and *H. americanum*, Kala et al. has demonstrated that lovastatin is present in higher concentrations in the biomass from in vitro cultures compared to the fruiting bodies (Figure 8). The mycelium of *H. coralloides* exhibited the highest concentration at 21.6 mg/100 g dry weight., while the lowest content in the mycelial biomass of *H. erinaceus* and *H. americanum* (5.81 mg and 2.64 mg in 100 g dry weight, respectively). Conversely, ergothioneine, was most abundant in the fruiting bodies of the analyzed mushroom species rather than in the biomass from in vitro cultures (Figure 8). Specifically, the concentration of ergothioneine in the fruiting bodies of *H. americanum* and H. erinaceus was 376 mg and 305 mg in 100 g dry weight., respectively. However, in the case of *H. coralloides*, the content of this compound decreased, ranging from 155 to 177 mg in 100 g dry weight. Importantly, the fruiting bodies of *H. americanum* were shown to be a superior source of biologically active compounds compared to the other investigated species [92].

Lovastatin is used to treat hypercholesterolemia, an activity it has due to its chemical structure, which inhibits 3-hydroxy-3-methylglutaryl coenzyme-A reductase in a highly competitive manner, blocking the binding of substrates and enzymes, thereby inhibiting cholesterol synthesis. In addition, its acidic and lactone structural conformations play an important role in lowering blood lipids. Lovastatin is also known for its efficacy in improving symptoms of depression, together with anti-inflammatory, neuroprotective, and anticancer effects [93].

In mammals, ergothioneine functions as a strong and non-toxic antioxidant agent, although its intake exclusively relies on diet. It is absorbed in the intestines and transported into cells and tissues by its specific transporter named Novel Organic Cation Transporter 1 (OCTN1). Ergothioneine accumulates mainly in the liver and erythrocytes, semen, eyes, and brain. The ability of ergothioneine to protect the cells comes from its reactive oxygen species scavenging activity, chelating, and other antioxidant pathways activation. It also has anti-radiation and anti-inflammatory activities. Oxidative stress and inflammation are key pathogenic factors in many diseases, including diabetes, as well as neurodegenerative, cardiovascular, and inflammatory bowel diseases. The accumulating studies support the preventive or therapeutic potentials of ergothioneine in a series of oxidative stress-related diseases through antioxidation [94].

#### 3.7.2. Herialpin A, Herialpin B, and Avenacein

**Figure 9:**
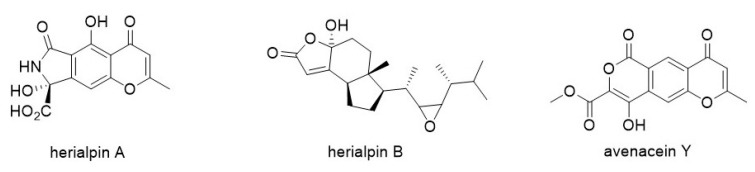
Chemical structure of heriaplins A and B and avenacein Y.

Li et al. described the isolation and structural determination of three new compounds derived from *Hericium alpestre*, known as herialpins A–B, and avenacein (Figure 9). The compounds were purified using medium-pressure chromatography over RP-18, followed by Sephadex LH-20, yielding 3.2 mg, 1.8 mg, and 2.1 mg from 2.9 g methanolic extract of fermented culture of *H. alpestre*, respectively. The structures were elucidated by 1D- and 2D-NMR data, ESI-MS, and X-ray crystallographic analysis [95].

The compounds were assayed for their cytotoxicity against three human tumor cell lines (A549, Hela, and HT-29). Avenacin Y showed selective positive activity against A549 and HT-29 cells with IC50 values of 15.1 and 20.1 μmol/L, respectively, while herialpins A–B showed a weak activity against these three tumor cell lines. The authors suggest that the pyrano[3,4-g]chromene-4,6-dione moiety in avenacein might be responsible for the stronger selective inhibitory activity.

## 4. Speculated Biosynthesis

### 4.1. Biosynthesis of Geranyl-Resorcinol-Type Metabolites

**Scheme 1:**
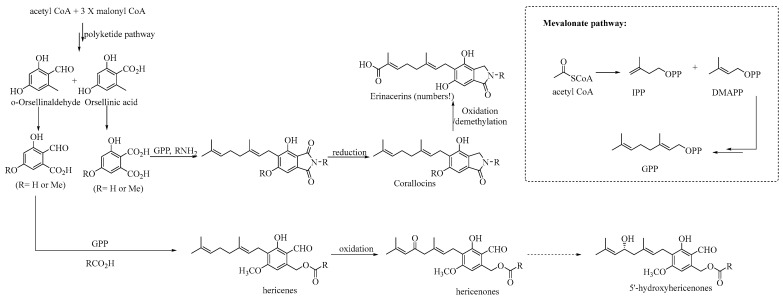
Proposed biosynthesis pathways geranyl-resorcinol-type metabolites.

The biosynthesis pathway of geranyl-resorcinol-type metabolites is thought to be generated from malonyl CoA and acetyl CoA through the hybrid polyketide-mevalonate pathways [96]. Orsellinic acid and o-orsellinaldehyde are proposed to be biosynthesized from the acetyl malonyl pathway, while geranyl pyrophosphate (GPP, C_10_) is produced from isopentyl pyrophosphate (IPP) and dimethylallyl pyrophosphate (DMAPP). Orsellinic acid and o-orsellinaldehyde are then suggested to undergo o-methylation and benzylic oxidation. Subsequently, both intermediates are assumed to undergo geranylation with GPP, along with either an amino acid or a fatty acid to produce isoindoline 1,3-dione and hericenes, respectively. Furthermore, hericenes may undergo oxidative metabolism on the geranyl side chain to produce hericenones, which may be followed by reductive metabolism to produce hydroxyhericenone. On the other hand, isoindoline 1,3-dione derivatives are transformed into corallocins (isoindoline derivatives) through reduction, followed by oxidation to produce erinacerin derivatives (Scheme 1) [34,57,97].

### 4.2. Biosynthesis of Erinacines Metabolites

**Scheme 2:**
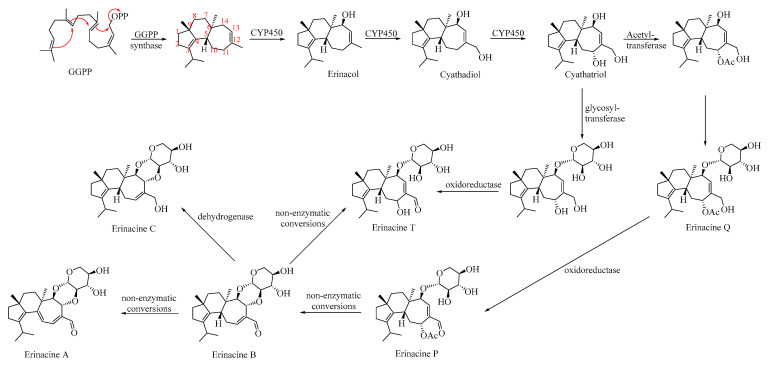
Proposed biosynthesis of erinacine derivatives.

Erinacines belong to the terpenoid family of natural products, derived from two 5-carbon precursors: IPP and DMAPP. These precursors undergo condensation head to tail under the action of isoprenyl diphosphate synthase, resulting in the formation of geranylgeranyl diphosphate (GGPP, C_20_), which cyclizes to form the cyathane 3,12-diene backbone (C_20_). For more detailed information about the transcriptome analysis and involved genes in biosynthesis, please refer to the cited publications [9,98,99,100]. Cytochrome P450 enzymes (CYPs) then sequentially hydroxylate C14, the methyl group of C12, and C11 to form cyathatriol. C11 of cyathatriol is subsequently acylated to form 11-O-acetyl cyathatriol, which is then catalyzed by xylosyltransferase to cyathane xyloside, known as erinacine Q. Afterwards, oxidoreductase catalyzes the conversion to erinacine P, which may undergo non-enzymatic conversion to form erinacine B, A, T, and C [101]. Alternatively, cyathatriol may undergo a glycosyltransferase reaction followed by an oxidoreductase reaction to produce erinacine T (Scheme 2) [98].

## 5. Conclusions

In this review, we made an effort to summarize the latest findings regarding secondary metabolites originating in *Hericium* ssp. and their bioactivity in mammalian models (Table 1). This vast array of compounds adds up to the already known rich plethora of functional compounds. While there is a growing understanding regarding the function of *Hericium* ssp.-derived compounds in mammalian models which include cell lines, mice, and humans, little is known about their cellular functionality in the mushroom itself.

Gene homology between different mushroom species and a conserved gene cluster as observed in the enzymes of erinacines metabolism could point towards a conserved functionality in the mushroom cell. Moreover, the localization of these metabolites and others to specific sections of the mushroom (for example, erinacines which are limited to the mycelium, and hericenones which are limited to the sporocarp) further supports the latter assumption and makes it tempting to identify a link between the compound’s chemical function in the mushroom tissue and its correlative affects in the mammalian bio-systems such as the neurotrophic, anti-inflammatory, and antioxidative activities described in the current review.

Identifying a specific target for the *Hericium* spp.-derived metabolites served as one of the approaches which aim at explaining its mechanism of activity. However, the functional redundancy that is witnessed in the erinacine, hericenone, and corallocin families could suggest that a wide approach examining overall gene regulation by whole mushroom extracts or specific metabolites is required.

It is expected that future studies will continue to identify novel bioactive metabolites in *Hericium* ssp. while further looking into their molecular mechanism. Surely, a better understanding of the compound’s chemical function and its correlation to its biofunctionality will promote the development of novel functional health foods, food supplements, and drugs aimed at supporting human health and treating various disorders.

## Figures and Tables

**Table 1:** Selected analyzed compounds with their corresponding biological activities.

Analyzed Compound	Sample Type	Biological Activity	Study Model	Reference
polysaccharides fraction HEP-C	*H. erinaceus* fruiting bodies	**Antioxidant potential**—scavenging activity Hypoglycemic potential—inhibitory effect on α-glycosidase and α-amylase	in vitro	[47]
polysaccharides fraction HEP-W	*H. erinaceus* fruiting bodies	**Immunomodulatory potential**—enhancing pinocytic and phagocytic capacity, increasing NO, IL-6, and TNF-α secretion	in vitro—RAW264.7 cells	[49]
polysaccharides fraction HEP	*H. erinaceus* fruiting bodies	**Antioxidant potential**—scavenging activity, reducing LDH and MDA production anti-apoptotic effect	in vitro—IPEC-J2 cells	[50]
polysaccharides fractions	*H. coralloides* fruiting bodies	**Antioxidant potential**—scavenging activities (ABTS, DPPH, hydroxyl, superoxide), protection against H_2_O_2_-induced cell death	in vitro—L929 cells	[51]
polysaccharides extract	*H. erinaceus* fruiting bodies	**Antioxidant potential**—decreased malondialdehyde levels, increased levels of the antioxidant enzymes (superoxide dismutase and catalase) in mice colon **Anti-inflammatory**—reducing pro-inflammatory factors (IL-6, IL-1β, and TNF-α), promoting the anti-inflammatory factor IL-10	in vivo—male C57BL/6 J mice	[52]
polysaccharides fraction PHEB	*H. erinaceus* fermented mycelia	**Neuroprotective and antioxidant activities**—improving cell viability of L-glutamate-exposed cells, improving AD-model mice cognitivity, alleviating amyloid-beta deposition and tau hyperphosphorylation, and antioxidant activity in the brain by the regulation of calcium homeostasis	in vitro—HT22 cells in vivo—APP/PS1 mice	[48]
hericioic acids A–G, hericiofuranoic acid	*H. flagellum* fruiting bodies	**Neurotrophic activity**—increased neurite outgrowths in PC12 cells	in vitro—PC12 cells	[62]
corallocin A	*H. coralloides* fruiting bodies	**Neurotrophic activity**—upregulation of NGF expression in astrocytoma cells followed by neurite outgrowth in PC12 cells	in vitro—PC12 cells, 1321N1 cells	[53]
	*H. erinaceus* fruiting bodies	Upregulation of NGF, BDNF, and SYP in glioma cells	in vitro—C6 cells, N2a cells	[54]
	*H. erinaceus* fruiting bodies	Elicitation of neurotrophic responses on hippocampal neuron culture	ex vivo—primary hippocampal neurons from E18 rat embryos	[56]
corallocin B	*H. coralloides* fruiting bodies	**Neurotrophic activity**—upregulation of BDNF expression in astrocytoma cells	in vitro—1321N1 cells	[53]
corallocin C	*H. coralloides* fruiting bodies	**Neurotrophic activity**—upregulation of NGF expression in astrocytoma cells followed by neurite outgrowth in PC12 cells, upregulation of BDNF expression	in vitro—1321N1 cells and PC12 cells	[53]
erinacerins Q, S, T	*H. erinaceus* fruiting bodies	**Antidiabetic potential**—inhibitory activity against PTP1B phosphatase and α-glucosidase	in vitro	[60]
erinacerin W	*H. erinaceus* mycelia	**Anti-inflammatory and neuroprotective activities**—alleviation of the LPS-activated BV-2 microglia-induced overexpression of IL-6, IL-1β, and TNF-α on neuroblastoma cells	in vitro—BV2 microglia- SH-SY5Y neuroblastoma co-culture	[58]
5′-hydroxyhericenes A–D	*H. erinaceus* fruiting bodies	**Antioxidant potential**—inhibition of xanthine oxidase activity	in vitro	[71]
hericenone L	*H. erinaceus* fruiting bodies	**Anti-cancerous potential**—against six human cancer cell lines	in vitro—SH-SY5Y, 1321N1, HCT-116, Caco-2, OVK18 and HeLa cells	[59]
hericenone Q	*H. erinaceus* fruiting bodies	**Anti-cancerous potential**—against human colorectal carcinoma and liver cancer cell lines	in vitro—Hep-G2 and HCT-116 cells	[74]
hericenone C and its deacylated derivative	*H. erinaceus* fruiting bodies	**Neurotropic and neuroprotective activities**—inducing BDNF expression and protection against H_2_O_2_-induced oxidative stress in astrocytoma cells	in vitro—1321N1 cells	[75]
N-dephenylethyl isohericerin, hericene A	*H. erinaceus* fruiting bodies	**Neurotrophic activity**—promoting extensive axon outgrowth and neurite branching in cultured hippocampal neurons	ex vivo—primary hippocampal neurons from E18 rat embryos	[56]
erinacine A	*H. erinaceus* mycelia	**Antioxidant and neuroprotective potential**—erinacine A-enriched extract impaired memory in an AD model of zebrafish	in vivo—wild type, short-fin strain zebrafish	[88]
	*H. erinaceus* mycelia	**Antioxidant and neuroprotective potential**—erinacine A-enriched extract recovered dopamine levels in MPTP-treated mice and lowered ROS levels in the brain, liver, and blood	in vivo—C57BL/6Narl male mice	[90]
	*H. erinaceus* mycelia	**Neuroprotective activities**—preventing cytotoxicity of neuronal cells and the production of ROS in vitro and in MPTP-treated mice	ex vivo—mouse N2a cells, mouse neuron substantia nigra cells in vivo—C57BL/6 mice	[40]
erinacine A, C, F	*H. erinaceus* mycelia	**Neurotrophic activity**—increased neurite outgrowths in PC12 cells **Anti-inflammatory and neuroprotective activity**—LPS-induced NO production inhibition in BV2 microglia cells	in vitro—PC12 cells, BV2 microglia cells	[89]
erinacine L	*H. erinaceus* mycelia	**Neurotrophic activity**—increased neurite outgrowths in PC12 cells **Anti-inflammatory and neuroprotective activity**—LPS-induced NO production inhibition in BV2 microglia cells	in vitro—PC12 cells, BV2 microglia cells	[89]
		Nitric oxide synthesis inhibition	in silico—molecular docking simulation	
erinacine S	*H. erinaceus* mycelia	**Neuroprotective activity**—preventing loss of oligodendrocytes and myelin during acute demyelination, and preserving myelin during chronic demyelination	ex vivo—oligodendrocyte cells from SD rat embryos in vivo—male SD rats	[81]
